# Comparative assessment of HPV, alcohol and tobacco etiological fractions in Algerian patients with laryngeal squamous cell carcinoma

**DOI:** 10.1186/s13027-018-0181-x

**Published:** 2018-03-13

**Authors:** Nora Kariche, Montserrat Torres Hortal, Samir Benyahia, Laia Alemany, Nabila Moulaï, Omar Clavero, Marleny Muñoz, Wahiba Ouahioune, Djamel Djennaoui, Chafia Touil-Boukoffa, Silvia de Sanjosé, Mehdi Bourouba

**Affiliations:** 1 Department of Cell and Molecular Biology, Team Cytokines and Nitric oxide synthases. Faculty of Biology, University Houari Boumediene USTHB, Bab-Ezzouar, Algiers, Algeria; 2 Oto-rhyno-laryngology Department, Mustapha Pacha Hospital, Algiers, Algeria; 3 Central Laboratory for Anatomopathology, Frantz fanon Hospital, Blida, Algeria; 4 Infections and Cancer Unit, Cancer Epidemiology Research Program, Catalan Institute of Oncology (ICO), Barcelona, Spain; 5 Bellvitge Institute of Biomedical Research (IDIBELL), Barcelona, Spain; 6 CIBER in Epidemiology and Public Health (CIBERESP), Barcelona, Spain

**Keywords:** Larynx carcinoma, HPV, Tobacco, Alcohol, Risk factor

## Abstract

**Background:**

Despite the increasing incidence of laryngeal squamous cell carcinoma (LSCC) in Algeria, scarce information is available on the importance of the preventable etiological factors which may drive the disease. Remarkably, a significant number of cases occur in nonsmoker and nondrinker patients; hence, suggesting that alternative risk factors, like Human papillomavirus (HPV), might be etiologically involved. To gain more insight on the risk factors associated with the disease in the country, we evaluated the etiological fraction of HPV in comparison to tobacco and alcohol intake in LSCC patients.

**Methods:**

To evaluate the etiopathologic fraction (EF) for HPV compared to history of tobacco and alcohol in LSCC, HPV DNA presence in 46 invasive and 3 non-invasive formalin-fixed paraffin-embedded laryngeal tumors was screened using the SPF10-DEIA-LiPA25 Assay. Demographic data and information related to exposure to the risk factors were gathered through interviewer-assisted questionnaires.

**Results:**

We observed that 40.8% of all LSCC cases were associated with smoking, 40.8% had combined tobacco and alcohol exposure history, and 14.3% did not show prior exposure to either risk factor. 1 out of 3 in-situ carcinoma cases was positive for HPV-6. HPV prevalence was null in the invasive tumors. HPV DNA was detected in 2.38% for all studied cases. 10.2% of LSCC patients did not associate with any of the studied risk factors.

**Conclusion:**

Here we show that HPV etiological fraction in LSCC Algerian patients is low and smoking and alcohol remain the principal etiopathologic risk for LSCC burden in Algeria.

## Background

Laryngeal cancer (LC) is the most common head and neck cancer (HNC). It accounted for 1.6% of all new cancer cases and 1.1% of deaths in 2012 worldwide [1]. The major histopathological form of these cancers consists of 95–98% laryngeal squamous cell carcinomas (LSCC) [2]. LSCC is a highly metastatic tumor, for which the 5-year survival rate at 10 years does not exceed 50% for stage IV [3]. The condition occurs principally between the fifth and the seventh decade of life and affects predominantly males (sex ratio 5:1 to 30:1) [4]. In Algeria, LSCC prevalence in men has significantly increased from 4.4% in 2009 to 5.6% in 2012, and the trend for the years to come predicts a steady increase to reach 6.3% by 2020; thereby reaching a 300% increase since 1986. Similarly, LSCC incidence increased from 1.7 to 6.4 per 10^5^ inhabitants between 1986 and 2010 [5, 6]. Considering the increasing impact of the disease on the Algerian population, it became important that improved knowledge on the exposure to the preventable risk factors of LSCC is acquired to ameliorate health policies and reduce the disease associated mortality.

Tobacco and alcohol are the main risk factors for LSCC. Both act as powerful forces driving epigenetic reprogramming and genetic instability to induce oncogenesis [4, 7, 8]. Tobacco has been incriminated as the most important risk factor for the development of the disease, with a relative risk varying from 1.5 to 9-fold [9]. Alcohol regular intake has been also implicated as an independent risk factor, and found to increase disease’s risk by 2 to 5-fold [10, 11]. Importantly, the combined effect of tobacco and alcohol has been shown to exceed the sum of the individual risks, with odds ratio reaching 177 for heavy drinkers and heavy smokers [11]. In Algeria, smoking and alcohol dependence have been lately notably higher in men compared to women (respectively, 27.1% vs. 1.7% in 2016; 1.4% vs. < 0.1 in 2010) and current reports have indicated an increase trend for tobacco and alcohol consumption in the country [12, 13]. Despite these numbers, no previous publication has ever evaluated the importance of these risk factors in LSCC patients.

Interestingly, approximately 5% of all LSCC showed to happen in nonsmoker and nondrinker patients, thus suggesting that alternative carcinogenic factors may induce LC [14]. In the last decade, human papillomavirus (HPV) infections have been associated with 22.1% of head and neck squamous cell carcinomas (HNSCC) [15] and have been related to 15–20% of those occurring in nonsmoking and nondrinking patients [16]. Marked differences in HPV attributable fraction in oral tumors have been shown to prevail between different populations and geographical regions [17–21]; these variations being due to heterogeneous testing procedures taking principally into account different number of biomarkers [15, 22]. Consequently, the previously over estimation of HPV associated fraction in the larynx, has been recently reevaluated to lower figures varying between 1.5% and 5.7%, depending on the biomarkers used (HPV DNA/mRNA/p16^INK4a^) [15, 17, 18]. To date, limited information is available on HPV burden in HNSCC in Algeria, and most of the available information on HPV infections is limited to cervical cancer, for which the prevalence for the general population has been estimate at 6.3% [23]. To the best of our knowledge, only a small cohort limited to 5 laryngeal tumors has been screened for HPV DNA, and for which, intriguingly, a null prevalence was reported [24].

To gain more insight on the prevalence of HPV infection in LSCC Algerian patients, a strict standardized protocol was applied to assess the etiological fraction of HPV compared to tobacco and alcohol in Algeria.

## Methods

### Specimen preparation

Tumors specimens were prospectively obtained from 58 patients, admitted between 2012 and 2016 at the Otolaryngology department of Algiers University Hospital Mustapha Pacha, upon informed consent. Cases were randomly recruited during two days per week; biopsies were performed on patients presenting a radiological report in favor of a laryngeal tumor, and fragments were used for the present study. The specimens were immediately fixed in formol (10%). Following fixation, the biopsies were rinsed twice in phosphate-buffered saline (PBS) and then manually dehydrated in ascending series of ethanol solutions (80%–100%, 1H). The samples were then cleared in xylene and embedded in paraffin.

Demographic (age, gender) and risk factors data (tobacco, alcohol and oral sexual practices) were gathered for the purpose of this study through an interviewer-assisted questionnaire led by the treating physician. This study was approved by the ethics committee of the Algerian National Agency for Research and Development in Health (ATRSS).

### Histopathological evaluation

The histopathological evaluation was performed for all collected specimens by expert pathologists based on the conventional classification of the world health organization (WHO). The type of infiltration and degree of differentiation of the tumors was evaluated, and the presence of HPV cytopathic effect (koilocytosis) was verified. Specimens with epithelial tumor in hematoxylin and eosin (HE)-stained sections were kept for analysis.

### HPV-DNA detection and genotyping

A sandwich sectioning method was used to prepare paraffin sections for HPV screening protocol as previously described [25]. In brief, four paraffin sections were obtained for each block. The outer sections served to confirm the histological diagnosis and the inner ones served for HPV detection. The microtome was cleaned after each sample sectioning and the blade was changed between the cuttings of each block. Gloves were regularly changed and blank paraffin block was systematically sectioned after each block to ensure no possibility of HPV DNA carryover between sectioning samples.

Paraffin sections of histologically confirmed cases were used to extract DNA. 250 μl of freshly prepared proteinase K solution (pH 8.0) was added directly to the paraffin sections and incubated at 70 °C until full digestion of the tissues. After proteinase K heat inactivation, HPV DNA detection was performed using SPF10-DEIA-LiPA25 version 1 system (Labo BiomedicalProducts, Netherlands). In brief, a 65-bp fragment of the L1 open reading frame of HPV was amplified employing the SPF10 consensus primers. Presence of HPV DNA was determined by DNA enzyme immunoassay (DEIA) using 10 μl of the amplified product that was hybridized to a mixture of conservative probes recognizing at least 54 HPV genotypes. Optical densities were read at 450 nm, and samples were categorized as HPV DNA negative, positive or borderline. Positive amplimers were genotyped using reverse hybridization line (LiPA_25_) that detects 25 high and low-risk HPV types (6, 11, 16, 18, 31, 33, 34, 35, 39, 40, 42, 43, 44, 45, 51, 52, 53, 54, 56, 58, 59, 66, 68, 70, and 74). Results were interpreted following manufacturer’s instructions. DNA quality of the samples was assessed by amplification of human tubulin gene with a specific set of primers. DEIA negative and tubulin positive samples were considered negative for HPV, while DEIA negative and tubulin negative samples were excluded from the HPV analysis.

### Statistical analyses

Statistical analyses were performed using GraphPad Prism software version 6.0.1. The Chi-square test was used to analyze the differences in proportion. *p*-values ≤0.05 were considered statistically significant.

## Results

### Description of the demographic and clinical characteristics of the studied cases

A total of fifty-eight cases of LC were prospectively obtained for analysis. Of these, nine were excluded after histological evaluation for absence of tumor tissue. Demographic and clinical description of the forty-nine evaluable cases is summarized in Table 1. The median age of the patients at the time of diagnosis was 66 years (range: 37–87). The male to female ratio was equal to 8.8:1.

**Table 1:** Descriptive characteristics of the patients. Demographic and clinical data

**Characteristics**	**Patients, n (%)**
**Gender**	
Male	44 (89.8)
Female	5 (10.2)
Median age at diagnosis (range)	66 (37–87)
Anatomic subsite	
Epiglottis	2 (4.1)
Glottis	29 (59.2)
Epiglottis/Glottis	13 (26.5)
Glottis/ Sub-glottis	2 (4.1)
Epiglottis/Glottis/Sub-glottis	3 (6.1)
Histological grade	
Carcinoma in situ	3 (6.1)
Invasive carcinoma	47(93.9)
- Conventional SCC	43(87.8)
- Basaloid	3 (6.1)

Of the 49 tumors, 29 (59.2%) were located in the glottis and two (4.1%) were located in the epiglottis. Tumors invading glottis and epiglottis or glottis and subraglottic areas were identified in 13 (26.5%) and two (4.1%) cases respectively. Three patients (6.1%) had tumors invading the three laryngeal regions. The majority of cases (93.9%) were diagnosed at invasive stage and only three tumors (6.1%) were in-situ carcinoma histological grade. Conventional type squamous cell carcinoma was the predominant histological type (87.8%).

### HPV etiologic fraction among LSCC is low

**Fig. 1:**
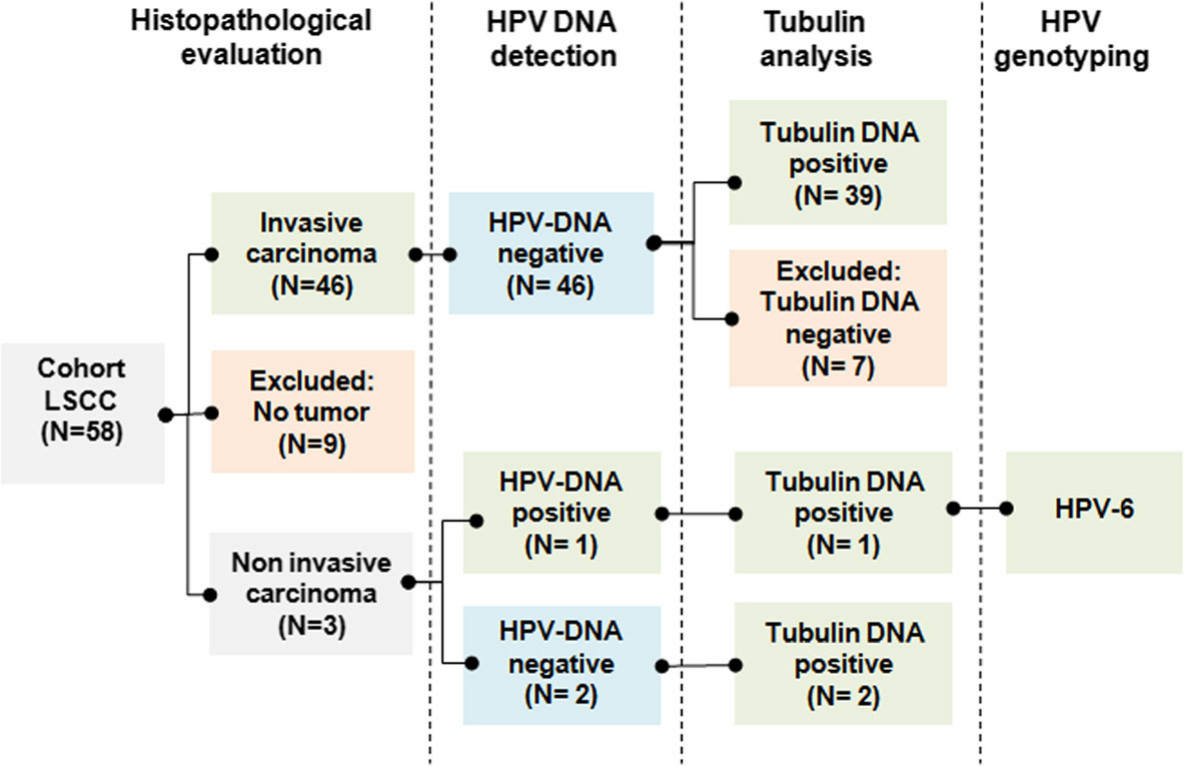
Chart of the work flow followed for the detection of HPV DNA in LSCC. The chart depicts the disposition of LC samples processing. DEIA-LiPA assay was performed to test for HPV DNA in 49 tumors. The tubulin gene was used as internal control

To screen for presence of HPV DNA, our samples were tested following the procedure outlined in Fig. 1. Insufficient DNA quality hampered HPV status analysis in seven invasive LSCC cases. Strikingly we observed that none of the remaining 39 analyzed invasive tumors showed to contain HPV DNA. Conversely, among the three diagnosed in-situ carcinoma cases, HPV DNA was detected only in one; therefore resulting in a prevalence of 2.38% (1/42 cases). Subsequent genotyping identified it as HPV6, (Fig. 1). Thus, HPV prevalence showed to be null among invasive LSCC, while concerning one in-situ carcinoma.

### Tobacco consumption alone or in conjunction with alcohol constitutes the major EF associating with LSCC

**Fig. 2:**
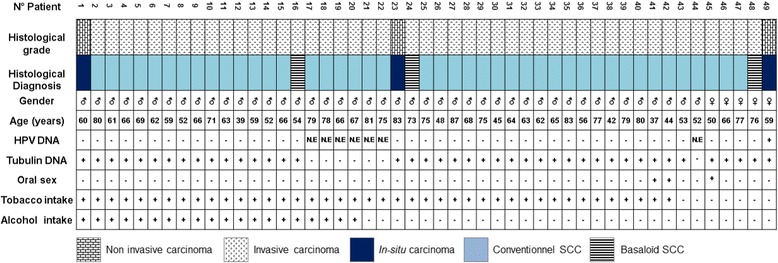
Individual histological and EF characteristics of the studied LSCC patients. The chart shows a stratification of the patients following ICO diagnosis, DEIA-LiPA assay results, and patients’ data gathered through interviewer-assisted questionnaires. N.E: Not Evaluable

To compare the etiopathologic fraction (EF) due to HPV with those due to the classical risk factors of LSCC, information on oral sex practice, tobacco, alcohol intake and HPV presence were confronted for each patient (Fig. 2). Patients’ stratification showed that 42 of them (85.7%) endorsed a history of smoking and 20 (40.8%) of alcohol exposure. Of these, respectively, 95.2% and 95%, presented invasive tumors. The combined rate of alcohol and tobacco exposure was 40.8%. Women declared themselves to be exposed only to second hand smoking, and thus only 7 patients (14.3%) fall into the nonsmoker-nondrinker category (Fig. 2). Patients’ stratification in function of the risk factors showed that 40.8% of LSCC cases would be due to tobacco alone and that alcohol intake was strictly associated with tobacco exposure.

**Fig. 3:**
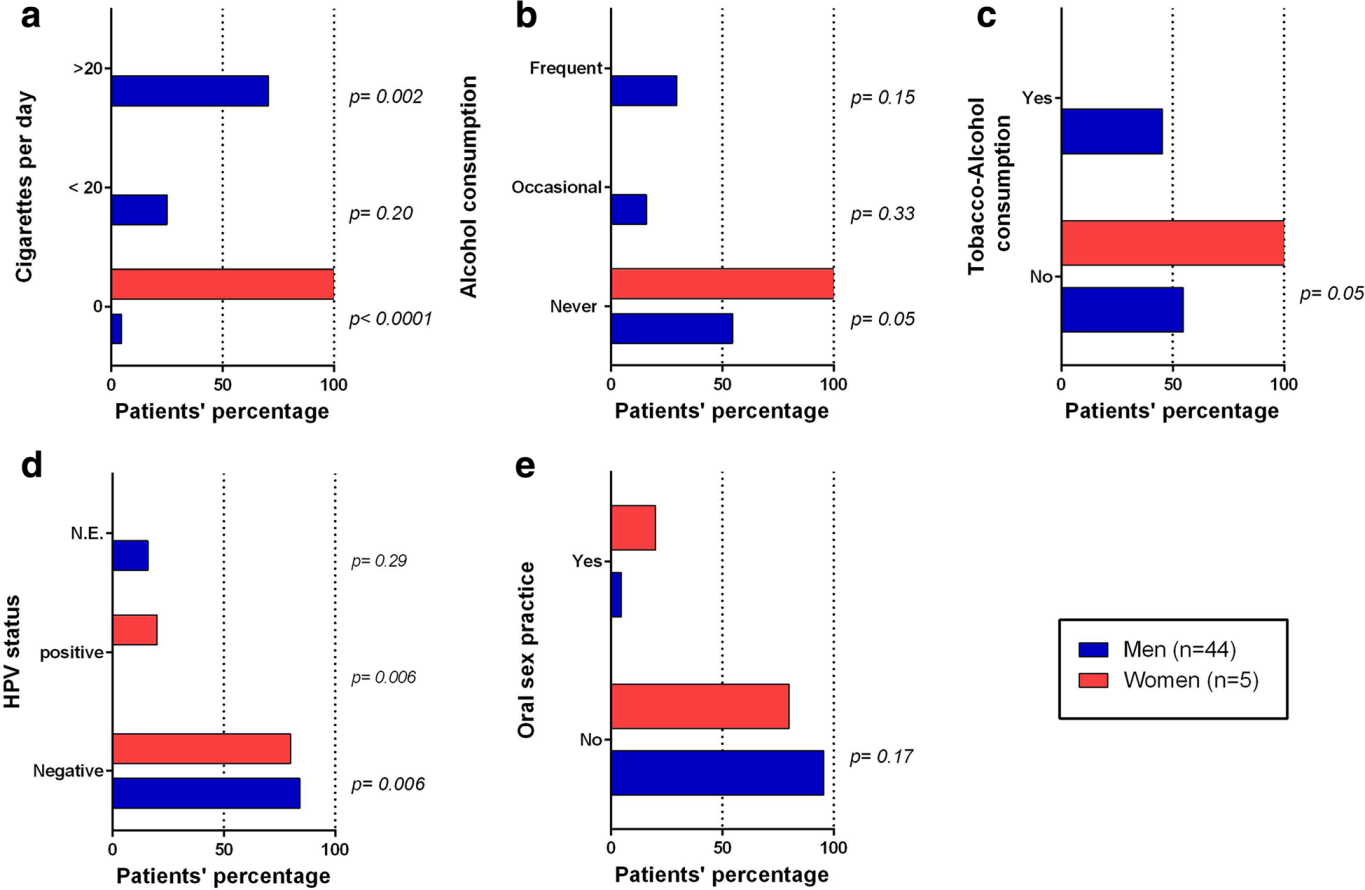
Risk factor exposure assessment by gender. Tobacco units were defined as number of cigarettes/day and alcohol exposure according to the frequency of drinks/week, HPV status was evaluated as previously described. Proportions were calculated among women (*n* = 5) and men (*n* = 44) and the χ2-test was used to analyze differences in proportion. *p* values ≤0.05 were accepted as statistically significant. N.E: Not Evaluable. Profiles by gender (expressed in percentage) of **a**) Cigarette consumption per day, **b**) Alcohol frequency consumption, **c**) Combined tobacco-alcohol consumption, **d**) HPV screening test result and **e**) Oral sex practice.

To evaluate the burden of the risk factors in the studied patients, tobacco units were defined as number of cigarettes/day; alcohol exposure was defined according to the frequency of drinks per week, and risk factors exposure was stratified based on the gender (Fig. 3). Among male patients, 70.45% were heavy smokers (> 20 cigarettes/day) and 25% were moderate smokers (≤20 cigarettes/day). Males endorsed a frequent alcohol consumption in 29.5% of cases and occasional in 15.9%. Therefore, our data show that tobacco consumption alone or combined with alcohol would constitute the highest etiologic fractions of LSCC in males.

### HPV prevalence in nonsmoker and nondrinker patients

As HPV related HNC has been described to be likely diagnosed in young, nonsmoker/nondrinker patients practicing oral sex [22], we next analyzed the profile of association existing between HPV status, oral sex practice, patient’s age and the smoking/drinking profile.

The single positive low risk HPV case was found in a nonsmoker-nondrinker woman aged 59, with no history of oral sex practice (Fig. 2). This result led to an observed significant difference in HPV status between women and men (*p = 0.005*) (Fig. 3D). We concluded then, that the etiopathologic fraction of HPV among nonsmokers and nondrinkers would equal 14.28% (1/7 cases) in the cohort and 20% among women.

Because oral sex practice may favor HPV transmission from infected women genitals to men upper aero-digestive tract and lead to oropharyngeal carcinogenesis in nonsmoking male partners [26], and as the prevalence of genital HPV in Algerian women reaches 6.3% [23], we extended our analysis to a possible association between LSCC risk factors and HPV for the 3 patients which reported having oral sexual activities (4.54% of men and 20% of women, *p = 0.17*) (Fig. 3E). Surprisingly, as no such associations were observed, we found that 10.2% of the patients (5/49 cases) did not associate with any of the studied risk factors. This result suggested that additional risk factors could be involved in LSCC etiology.

## Discussion

Similarly to the rest of the world, Algeria has witnessed during the last 30 years a dramatic increase in LSCC incidence rate [5]. This epidemiologic transition prompted the scientific community to investigate its preventable causes, and to explore HPV as an emerging alternative risk factor of oral carcinogenesis. Contrarily to oropharyngeal carcinoma, the role of the virus in LC remains to date intriguing, particularly when considering the various meta-analyses which have estimated the prevalence of HPV in LC [25] and its frequency in nonsmoking and nondrinking LC patients [16]. Despite the global interest for the association, no prior study has ever conducted a comparative assessment of HPV, tobacco and alcohol as risk factors of LSCC in the country.

Here, we estimated the EF for HPV in LSCC at 2.38%. Our observation is in agreement with the most recent evaluations which showed that the EF for HPV in LC is generally low in comparison to other HNC sites [15, 17]. Interestingly, this finding places Algeria, among the countries where LC would be the less affected by HPV infections [18, 25]. Indeed, several authors have described higher HPV prevalence for Latin-America compared with Western-Africa, Southern-Europe, USA and Southwestern-Asia [17, 19–21]. When taking into account the impact that the screening techniques may exert on HPV detection [17, 19, 25], we observed that HPV prevalence in Algeria was inferior to that described for the larynx in the world [17, 18]. At the opposite, it showed to be superior to that found in India (1.7%) [27]. These differences would likely result from the possible influence that the age, the genetics and the environment of a patients may exert on viral transmission and persistence in an infected tissue [25, 28]. Therefore, it is possible that the advanced age of our study group may have constituted a possible limit to HPV detection, in particular considering that HPV would likely be diagnosed in younger adults under the age of 50 [28–30]. Another reason for the low HPV prevalence in our cohort, would probably be due to the low frequency of oral sex practice among aged patients, and especially among males, which have shown by others to carry the highest risk of oral infection [31]. Exploring the differences in oral sex practice in men, might clarify the observed differences. By contrast, we observed a higher prevalence of oral sex practice and HPV infection among women.

Considering our observation, that the single HPV-LC positive case was found in a women, the recent demonstrations that oral HPV would be frequently detected in patients with HPV cervical lesions [32], and the important rate of genital HPV infections in female in Algeria [23], it is important that next studies focus on determining the rate of association existing between cervical HPV lesions and HPV prevalence in LSCC patients. Also, future explorations should focus on male partners of women with HPV cervical lesions, as they may have a significantly greater incident rate of HPV related HNC [28, 33]. Therefore, we believe that a focus should be put next, in particular, on examining the prevalence of HPV infections among sexual partners for which females have been diagnosed with HPV infection(s), and consider their follow-up to evaluate the HPV related risk of carcinogenesis.

In this study, we also observed that, while among the invasive LSCC cases HPV prevalence was null, 1 out of 3 in-situ carcinoma cases were positive for HPV6. These findings are in agreement with reports from Castellsague et al. (2016), which described HPV6 as figuring among the most frequently detected HPV genotypes associating with LC [17]. It is noteworthy that Villagómez-Ortíz and al. (2016) proposed that HPV infections would tend to be diagnosed at early stages of LSCC progression, in particular among laryngeal papillomatosis (LP) with severe dysplasia cases [29]. LP is a benign tumor, frequently associating with HPV6 or HPV11, which shifts in 1–4% cases towards malignancy [34]. Despite this association, several studies pointed out that HPV DNA positivity in LC would not prove the viral oncogenicity, as it may only be coincidental with a transient infection which happens also to occur in 11.2% normal laryngeal tissues [35]. Therefore, complementary screenings including amplification of the viral E6 transcript and interrogation of the specific genetic alterations induced by HPV infection should be conducted to conclude on the carcinogenic aspect of the viral infection [36, 37].

Our observation that LSCC occurs in absence of HPV, enforces the idea that tobacco and alcohol may be central to laryngeal carcinogenesis. Indeed, from a total of 49 patients, 85.7% were active smokers, 40.8% were alcohol drinkers and 40.8% had a mixed profile. Considering the heavy frequency of smoking (> 20 cigarettes/day) observed in our patients, the exposure to tobacco seems to constitute the principal cause of the disease. This idea is further supported by the reports indicating a wider access to tobacco in the society over alcohol, as indicated by the recent estimates indicating that 25.3% of men, 0.3% of women and 18% of boys would daily use tobacco [38] and WHO data reporting a total alcohol consumption per Algerian capita (> 15 years of age) under 2.5 L of pure alcohol in 2015 [39]. From the analysis of the risk factors associating with the nonsmoker/nondrinker profile, we observed a low prevalence of the viral etiology (14.28%). Due to rarity of both events, this number should be confirmed on a much larger cohort also including younger patients. Indeed, substantial findings have indicated that HPV-induced HNC would rather occur in younger patients with frequent oral sex practice [22]. Thus, considering the possible behavioral changes that may occur in the population, we suggest to keep on evaluating the risk factors implicated in LSCC.

By contrast to the literature, we found that 10.2% of the tumors occurred in patients showing no prior exposure to any of the studied risk factors; this observation exceeded by two folds the generally observed rates [14]. Hence, we hypothesize that unexplored factors may particularly contribute to LSCC development in Algeria. To get a better insight on the matter, future studies should explore, in priority exposure to second-hand smoking and to the known dietary and environmental carcinogens like salt-preserved food and coal dust for their likelihood to increase the risk of LSCC [40–42]. As well as, endogenous factors like gastroesophageal reflux disease which could act as a promoting factor in the malignant transformation of the normal mucosal surface of the larynx [43]. Exploring the recently discovered gene variants of susceptibility to the condition, like in Mdm2 (Mouse double minute 2) and BCL11 (B-cell lymphoma), and those incriminated specifically to have no interaction with drinking and smoking, in HLA (human leukocyte antigen) region 6p21, FADS1 gene (fatty acid desaturase 1) and tumor suppressor TBX5 (T-box 5) genes, should be analyzed to quantify the inherited risk to develop LSCC in nondrinkers and nonsmokers, and provide a rational ground to anticipate and prevent the disease in the population [44–46].

## Conclusion

In summary, our data suggest that HPV infection would have little impact on the development of LSCC, while tobacco and alcohol, would represent its major preventable risk factors. Considering the growing accessibility to tobacco and alcohol for the youngest, it is probable that the disease burden will remain high for the next decades in the country. Although our findings need to be confronted to larger case series, they constitute, to the best of our knowledge, the first rational arguments for the national health authorities to consider policies to reduce tobacco and alcohol consumption to decrease LSCC prevalence in Algeria and warrant the evaluation of the inherited risk to develop LSCC in the general population.

## Abbreviations

ATRSS: Algerian national agency for research and development in health
BCL11: B-cell lymphoma
DEIA: DNA enzyme immunoassay
DNA: deoxyribonucleic acid
EF: etiopathologic fraction
FADS1: fatty acid desaturase 1
HE: hematoxylin and eosin
HLA: human leukocyte antigen
HNC: head and neck cancer
HNSCC: head and neck squamous cell carcinomas
HPV: human papillomavirus
ICO: Institute Catalan of oncology
LC: laryngeal cancer
LP: laryngeal papillomatosis
LSCC: laryngeal squamous cell carcinoma
Mdm2: Mouse double minute 2
mRNA: messenger Ribonucleic acid
PBS: phosphate-buffered saline
TBX5: T-box 5
USA: United States of America
Vs: versus
WHO: world health organization
